# Reviewer acknowledgement 2014

**DOI:** 10.1186/s13073-015-0139-1

**Published:** 2015-02-20

**Authors:** Rebecca Furlong

**Affiliations:** BioMed Central, 236 Gray’s Inn Road, London, WC1X 8HB UK

## Abstract

The editors of *Genome Medicine* are extremely grateful for the time, hard work and support of all our reviewers, and would like to thank everyone who contributed to the journal in Volume 6 (2014).

**Gosse Adema**

Netherlands

**John Aitchison**

USA

**Patrick Aloy**

Spain

**Christopher Amos**

USA

**Miguel Andrade-Navarro**

Germany

**Marcella Attimonelli**

Italy

**Thomas Aune**

USA

**Fabio Bagnoli**

Italy

**Vikas Bansal**

USA

**Anne-Laure Banuls**

France

**Fredrik Barrenas**

USA

**Anastasia Baryshnikova**

USA

**Arnold Bayer**

USA

**Terri Beaty**

USA

**Stephan Beck**

UK

**Roderick Beijersbergen**

Netherlands

**Gillian Bell**

USA

**John Belmont**

USA

**Mikael Benson**

Sweden

**Tushar Bhagat**

USA

**Dwaipayan Bharadwaj**

India

**Leslie Biesecker**

USA

**Ewan Birney**

UK

**Hans Bjornsson**

USA

**Pascal Borry**

Belgium

**Anne Bowcock**

UK

**Hassan Brim**

USA

**Kyle Brothers**

USA

**Alistair Brown**

UK

**Ken Buetow**

USA

**John Bunge**

USA

**Wylie Burke**

USA

**Paul Cairns**

USA

**Monica Campillos**

Germany

**Hannah Carter**

USA

**Don Chalmers**

Australia

**Rong Chen**

USA

**Christophe Chevillard**

France

**Laurent Chiche**

France

**Charles Chiu**

USA

**Dmitriy Chudakov**

Russian Federation

**Eric Collisson**

USA

**Kenneth Cornetta**

USA

**Emmanuel Cornillot**

France

**Manuel Corpas**

UK

**Hector Corrada Bravo**

USA

**Fergus Couch**

USA

**Sally Coulthard**

UK

**Jeffrey Craig**

Australia

**Petr Danecek**

UK

**Paul de Bakker**

Netherlands

**Michael DeGiorgio**

USA

**Joaquin Dopazo**

Spain

**Wolfgang Doppler**

Austria

**Yali Dou**

USA

**Joel Dudley**

USA

**David Duffy**

Australia

**Eric Duncavage**

USA

**Paul Edwards**

UK

**Sol Efroni**

Israel

**Nathan Ellis**

USA

**Rachel Ellsworth**

USA

**José Manuel Entenza**

Switzerland

**David Erle**

USA

**Daniel Falush**

Germany

**Cristiano Fava**

Italy

**Andrew Feinberg**

USA

**Jorge Ferrer**

UK

**Gary Firestein**

USA

**James Flanagan**

UK

**Florian Fricke**

USA

**Holger Fröhlich**

Germany

**Laura Ines Furlong**

Spain

**Alison Gammie**

USA

**Jennifer Gardy**

Canada

**Lana Garmire**

USA

**Mathew Garnett**

UK

**Elliot Gershon**

USA

**Phil Giffard**

Australia

**Lyn Gilbert**

Australia

**Jill Goldman**

USA

**Tom Gonda**

Australia

**Abel Gonzalez-Perez**

Spain

**Assaf Gottlieb**

USA

**Ramaswamy Govindan**

USA

**Lloyd Graham**

Australia

**Kristi Graves**

USA

**Celia Greenwood**

Canada

**Joshua Grill**

USA

**Robert Gruetzmann**

Germany

**Loic Guillot**

France

**Juergen Haas**

UK

**Christopher Haiman**

USA

**Alison Hall**

UK

**Barry Hall**

USA

**Sridhar Hannenhalli**

USA

**Toshiro Hara**

Japan

**Joshua Hare**

USA

**Reuben Harris**

USA

**Peter Harvey**

Australia

**Rick Twee Hee**

Singapore

**Gert Helgesson**

Sweden

**Chengtao Her**

USA

**Julian Hiscox**

UK

**Margret Hoehe**

Germany

**Robert Hoehndorf**

UK

**Noah Hoffman**

USA

**Heidi Howard**

Sweden

**R Stephanie Huang**

USA

**Chad Huff**

USA

**Russell Hyde**

UK

**Andrew Jarnicki**

Australia

**Olli Kallioniemi**

Finland

**Yae Kanai**

Japan

**Le Kang**

USA

**Natarajan Kannan**

USA

**Arek Kasprzyk**

Italy

**Karl Kelsey**

USA

**David Kelvin**

Canada

**Paul Kemp**

UK

**Hans Kestler**

Germany

**Halil Kilicoglu**

USA

**Dokyoon Kim**

USA

**Thomas Kislinger**

Canada

**Eric Klee**

USA

**Steven Kleinstein**

USA

**Jo Knight**

Canada

**Bartha Knoppers**

Canada

**Barbara Koenig**

USA

**Sek Won Kong**

USA

**Rui Kuang**

USA

**Martin Larsen**

Denmark

**Jin Soo Lee**

South Korea

**Peter Lee**

USA

**Christina Leslie**

USA

**Xia Li**

China

**Liming Liang**

USA

**Mathias Lichterfeld**

USA

**Daniel Lim**

USA

**Rune Linding**

Denmark

**John LiPuma**

USA

**Yang Liu**

USA

**Nuria Lopez-Bigas**

Spain

**Simon Lovestone**

UK

**Chris Lowrey**

USA

**Robert Lyle**

Norway

**Jian Ma**

USA

**Shuangge Ma**

USA

**Renaud Mahieux**

France

**Gathsaurie Malavige**

Sri Lanka

**Natalia Malsev**

USA

**Subhrangsu Mandal**

USA

**Kenneth Mandl**

USA

**Demetrius Maraganore**

USA

**Anthony Marinaki**

UK

**Florian Markowetz**

UK

**John Martens**

Netherlands

**Manuel Mayr**

UK

**Peter Meikle**

Australia

**Alexander Mellmann**

Germany

**George Michailidis**

USA

**Jason Moffat**

Canada

**Karen Mohlke**

USA

**Stephen Montgomery**

USA

**Servaas Morre**

Netherlands

**David Morris**

UK

**Kenta Nakai**

Japan

**Andre Nantel**

Canada

**Petr Nazarov**

Luxembourg

**Deborah Neklason**

USA

**Pauline Ng**

Singapore

**Robert Nussbaum**

USA

**Thomas Otto**

UK

**Massimiliano Pagani**

Italy

**Tom Palmer**

UK

**Anna Panchenko**

USA

**William Pao**

USA

**Nickolas Papadopoulos**

USA

**Choon-Sik Park**

South Korea

**Joel Parker**

USA

**Justin Paschall**

UK

**Andrea Patenaude**

USA

**Robert Paulson**

USA

**Sharon Peacock**

UK

**Christoph Plass**

Germany

**Sharon Plon**

USA

**Manuel Portoles**

Spain

**Jonathan Powell**

USA

**Barbara Prainsack**

UK

**Graham Pryor**

UK

**Arshed Quyyumi**

USA

**Sridharan Raghavan**

USA

**Simon Ramsden**

UK

**Benjamin Raphael**

USA

**Heidi Rehm**

USA

**Juri Reimand**

Canada

**Mary Relling**

USA

**Owen Rennert**

USA

**Markus Ringnér**

Sweden

**Janet Risk**

UK

**Manuel Rivas**

UK

**Peter Robinson**

Germany

**Doreen Robinson**

USA

**Noah Rosenberg**

USA

**M Geoffrey Rosenfeld**

USA

**Charles Rotimi**

USA

**Sameek Roychowdhury**

USA

**Gregory Ryslik**

USA

**Julio Saez-Rodriguez**

UK

**Paul Schofield**

UK

**Annemie Schols**

Netherlands

**Ekkehard Schütz**

Germany

**Dominik Seelow**

Germany

**Julie Segre**

USA

**Shigeki Sekine**

Japan

**Sohrab Shah**

Canada

**Sonia Shah**

Australia

**Amitabh Sharma**

India

**Chad Shaw**

USA

**Valery Shestopalov**

USA

**Jaekyu Shin**

USA

**So-Youn Shin**

UK

**Joshua Shulman**

USA

**Henner Simianer**

Germany

**Richard Simon**

USA

**Richard Simpson**

Australia

**Marina Sirota**

USA

**Heather Skirton**

UK

**Pamela Sklar**

USA

**Jeffrey Skolnick**

USA

**Margaret Sleeboom-Faulkner**

UK

**Michael Snyder**

USA

**David Solit**

USA

**Mingzhou Song**

USA

**Qing Song**

USA

**Erik Sonnhammer**

Sweden

**Richie Soong**

Singapore

**Boe Sorensen**

Denmark

**Therese Sørlie**

Norway

**Nicole Souren**

Germany

**Allan Spigelman**

Australia

**Avrum Spira**

USA

**Marja Steenman**

France

**Oliver Stegle**

UK

**Barbara Stranger**

USA

**Karsten Suhre**

Qatar

**Patrick Sullivan**

USA

**Michael Surette**

Canada

**Jawahar Swaminathan**

UK

**Kate Sweeny**

USA

**Alan Tait**

USA

**Patrick Tang**

Canada

**Hugh Taylor**

Australia

**John Tentler**

USA

**Andrew Teschendorff**

UK

**Herve Tettelin**

USA

**Ali Torkamani**

USA

**Zlatko Trajanoski**

Austria

**Johan Trygg**

Sweden

**Koji Tsuda**

Japan

**Benjamin Tycko**

USA

**Svitlana Tyekucheva**

USA

**Susan Vadaparampil**

USA

**Alfonso Valencia**

Spain

**Alejandro Vallejo**

Spain

**Anne Van den Broeke**

Belgium

**Russell Van Gelder**

USA

**Fabio Vandin**

Denmark

**Jeffrey Varner**

USA

**Kenneth Vernick**

France

**Peter Visscher**

Australia

**Daniel Von Hoff**

USA

**Mia Wadelius**

Sweden

**Gerard Wagemaker**

Netherlands

**Wolfgang Wagner**

Germany

**Junwen Wang**

China

**Kai Wang**

USA

**Yibo Wang**

China

**Rene Warren**

Canada

**Corey Watson**

USA

**Peter Watson**

Canada

**Karen Weck**

USA

**Chia-Lin Wei**

USA

**Michael Weiss**

USA

**Harm-Jan Westra**

Netherlands

**Malcolm Whiteway**

Canada

**Marc Williams**

USA

**Ingrid Winship**

Australia

**Ellen Wright Clayton**

USA

**Wei Wu**

USA

**Mark Yandell**

USA

**Martin Yarmush**

USA

**Deborah Zarin**

USA

**Julia Zeitlinger**

USA

**Dongxiao Zhu**

USA

**Qian Zhu**

USA

**Hub Zwart**

Netherlands

